# Tailoring Metal Phthalocyanine/Graphene Interfaces for Highly Sensitive Gas Sensors

**DOI:** 10.3390/nano15090691

**Published:** 2025-05-03

**Authors:** Daniele Perilli, Alberto Maria Rizzi, Cristiana Di Valentin

**Affiliations:** Department of Materials Science, University of Milano-Bicocca, Via R. Cozzi 55, I-20125 Milano, Italy

**Keywords:** graphene, metal phthalocyanine, NH_3_, NO_2_, gas sensing, density functional theory calculations

## Abstract

Developing novel gas-sensing materials is critical for overcoming the limitations of current metal oxide semiconductor technologies, which, despite their widely commercial use, require high operating temperatures to achieve optimal performance. In this context, integrating graphene with molecular organic layers provides a promising platform for next-generation gas-sensing materials. In this work, we systematically explore the gas-sensing properties of metal phthalocyanine/graphene (MPc/Gr) interfaces using density functional theory calculations. Specifically, we examine the role of different MPcs (FePc, CoPc, NiPc, and CuPc) and Gr doping levels (p-doped, neutral, and n-doped) in the detection of NH_3_ and NO_2_ molecules, used as representative electron-donor and -acceptor testing gases, respectively. Our results reveal that a p-doped Gr is necessary for NH_3_ detection, while the choice of metal cation plays a crucial role in determining sensitivity, following the trend FePc/Gr > CoPc/Gr > NiPc/Gr, with CuPc/Gr exhibiting no response. Remarkably, FePc/Gr demonstrates sensitivity down to the limit of a single NH_3_ molecule per FePc. Conversely, NO_2_ detection is possible under both neutral and n-doped Gr, with the strongest response observed for n-doped FePc/Gr and CoPc/Gr. Crucially, we identify the d_z2_ orbital of the MPc as a key factor in mediating charge transfer between the gas molecule and Gr, governing the electronic interactions that drive the sensing response. These insights provide valuable guidelines for the rational design of high-sensitivity graphene-based gas sensors.

## 1. Introduction

Gas-sensing materials are key components of sensing devices used in a wide range of applications, from traditional fields such as environmental monitoring to cutting-edge fields like food quality control and medical diagnostics [1,2,3,4]. In the latter case, breath analysis, also known as breathomics, is an emerging technique for the early and non-invasive diagnosis of diseases, based on the detection of specific biomarker molecules in exhaled breath [2,3]. When the concentration of these molecules exceeds a certain threshold, it can serve as an early warning signal for potential health issues.

For practical applications, sensors must combine high sensitivity, selectivity, stability, and fast response while operating at room temperature (RT) to enable integration into portable and wearable devices [2,4,5]. However, current state-of-the-art technologies rely primarily on metal oxide semiconductors (MOSs) [6], which, despite their high sensitivity and low detection limits, suffer from poor gas selectivity and require high operating temperatures (100–300 °C) [7]. This leads to large energy consumption [8], higher signal drift, and a shortened lifespan due to thermal degradation. As their intrinsic sensing mechanism is thermally activated [9], there is an urgent need for next-generation materials capable of operating efficiently at RT.

In this regard, two-dimensional (2D) materials, such as graphene (Gr) [10,11], transition metal dichalcogenides [12,13], black phosphorus [14,15], and hexagonal boron nitride [16], offer a promising alternative for highly sensitive gas sensing applications. These materials, whether used individually or in combination, exhibit unique electronic structures, large surface areas, and tunable properties that make them ideal for sensing [10,11]. In particular, Gr, a single layer of C atoms arranged in a honeycomb lattice, has emerged as one of the most promising 2D materials for gas sensing applications [17,18,19,20]. As the first isolated and most extensively studied 2D material, Gr benefits from well-established and scalable synthesis and functionalization methods, allowing for cost-effective fabrication with atomic-scale precision [21]. Its 2D structure provides a large specific surface area, maximizing exposure to gas molecules and thereby enhancing the sensor’s response. Moreover, Gr exhibits a unique combination of physical properties, including high thermal conductivity, excellent charge carrier mobility at RT, mechanical strength, flexibility, and optical transparency. From an electronic point of view, its unique electronic properties, characterized by a conical intersection of the valence and conduction bands at the Fermi level, enable high charge carrier mobility and exceptional electrical conductivity. These features make Gr an electronically low-noise material with a high signal-to-noise ratio [20], making it an ideal platform for highly sensitive gas detection at RT. This also allows for integration into portable, wearable, and low-power sensing devices. For instance, Schedin et al. [20] were the first to experimentally demonstrate Gr’s extraordinary ability to detect even a single NO_2_ molecule at RT. Following this result, subsequent studies explored Gr’s response to other gases at RT. Chen et al. [22] showed that Gr is sensitive to O_2_, while Yavari et al. [23] investigated its performance in NH_3_ and NO_2_ atmospheres, reporting detection limits as low as 500 ppb and 100 ppb, respectively.

However, despite its high sensitivity at RT, Gr suffers from a slow recovery time, often requiring annealing at 200 °C to fully restore its functionality [20,23]. To address this limitation and further enhance its sensitivity and selectivity, research has moved from pristine to chemically and physically modified forms of Gr [24,25]. For instance, nanolithography-treated Gr was found to exhibit a rapid response, quick recovery, and high sensitivity to various gas molecules [26]. This improvement is attributed to the contaminations introduced by the physical treatment, which increase carrier scattering and introduce adsorbant sites for concentrating analyte molecules. Similarly, boron doping was found to boost Gr’s sensitivity by 27 and 105 times to reach detection limits of 95 and 60 ppb, respectively, for NO_2_ and NH_3_ detections [27].

Among the various functionalization strategies developed to date, a particularly valuable approach for gas sensing involves interfacing graphene with (metallo)organic molecules [28,29] that can interact either through covalent bonds (grafting) or weak interactions, such as π-π stacking. Covalent functionalization alters Gr’s carbon hybridization from sp^2^ to sp^3^, while non-covalent interactions can modulate the charge carrier concentration, shifting Gr from a neutral to either a p-type doped (positively charged) or an n-type doped (negatively charged) state via net charge transfer. Additionally, Gr electronic doping can be achieved by (i) supporting Gr on specific substrates that can donate (n-doping) or accept (p-doping) electron density to/from Gr [30,31]; (ii) using substitutional dopants like boron and nitrogen in the Gr lattice, which induce p-doping and n-doping [32], respectively; and (iii) exposing Gr to air, which leads to p-doping due to partial oxidation by O_2_ molecules [28]. This is especially relevant for gas sensing, as the charge carrier concentration directly affects the adsorption of gas molecules on Gr’s surface [33]. Moreover, non-covalent functionalization has the advantage of preserving Gr’s electron transport properties. Additionally, using Gr as the substrate offers the unique capability of controlling the distribution of molecules [34] or substitutional dopants [35], ensuring their controlled arrangement on or within the lattice.

Within this framework, metal phthalocyanines (MPcs)—organic macrocyclic molecules coordinating metal divalent cations (M^2^⁺)—have been extensively studied for their ability to form non-covalent heterointerfaces with Gr, ranging from sub-monolayers to thick films [36,37,38,39,40,41,42]. MPcs exhibit tuneable chemical, electronic, and optical properties, making them highly suitable for applications in photocatalysis, electrocatalysis, spintronics, and gas sensing [43,44,45,46,47]. Notably, the choice of the metal cation plays a crucial role in shaping the molecule’s electronic properties, thus enabling their precise tuning [48].

Along this research line, we recently combined theoretical simulations and experiments to investigate the interface formed by thermally depositing nickel phthalocyanine (NiPc) molecules onto a p-doped Gr monolayer, aiming to develop a chemiresistive gas sensor for NH_3_ and NO_2_ detection [49,50]. Our findings revealed that this system exhibits a strong response to both gases at RT, with the Ni d_z2_ orbital playing a pivotal role in mediating the electronic communication between the target gases and the graphene layer.

These results raise two key open questions: (i) To what extent and how does the choice of metal cation influence the system’s sensitivity? (ii) To what extent and how do the type (holes or electrons) and amount of charge carriers in electronically doped Gr affect the sensitivity?

Addressing these questions is essential for the rational design of MPc/Gr-based sensors, enabling the precise tailoring of their properties for optimal performance.

In this work, we employ density functional theory (DFT) calculations to investigate the structural and electronic properties of electronically doped Gr functionalized with various MPcs for gas sensing applications. We focus on Fe, Co, Ni, and Cu as central metal cations, given their widespread use in metal phthalocyanine-based interfaces produced through deposition techniques. To assess the sensing capabilities, we consider NH_3_ and NO_2_ as model gases, representing typical electron-donor and electron-acceptor gases, respectively. Building on previous findings [50], our goal is to (1) examine how the metal cation affects the electronic properties and gas sensing sensitivity of the MPc/Gr system, and (2) investigate how Gr’s charge state, i.e., its charge carrier doping level, influences the electronic behavior and the overall sensitivity.

Understanding these aspects can help the design of more efficient and rational Gr-based gas sensors for applications requiring high sensitivity to NH_3_ and NO_2_ at RT. Such applications include environmental monitoring, where these gases are toxic pollutants; food quality tracking, as they are often produced by spoiled food; and breathomics, as they serve as biomarkers for various diseases.

## 2. Computational Methods

The spin-polarized DFT + U calculations were performed using the plane-wave-based Quantum ESPRESSO package (QE) [51,52]. The ultrasoft pseudopotentials were employed to describe the electron–ion interactions, with H (1s), C (2s, 2p), N (2s, 2p), O (2s, 2p), Fe (4s, 3d), Co (4s, 3d), Ni (4s, 3d), and Cu (4s, 3d) considered valence electrons [53]. Energy cutoffs of 46 and 326 Ry (for kinetic energy and charge density expansion, respectively) were adopted for all calculations. The Perdew–Burke–Ernzerhof (PBE) functional was used for electron exchange–correlation [54], with van der Waals interactions included via semiempirical corrections using the DFT-D3 formalism [55]. Dispersion forces are particularly important for accurately modeling the interactions between Gr π states and aromatic systems. In this context, the D3 method provides a cost-effective and reliable approach.

To accurately model the electronic structure of MPc molecules, a Hubbard U correction was applied to the metal d-orbitals. This approach offers a good compromise between computational cost and accuracy and has been widely adopted for MPc systems. Based on values reported in the literature [56,57], U parameters of 5 eV for Fe, 4 eV for Co, 5 eV for Ni, and 4 eV for Cu were applied to the d-orbitals in both gas-phase and Gr-supported MPc molecules.

During geometry optimization, all atoms were allowed to relax using a convergence criterion of 0.026 eV/Å for forces and 10^−6^ Ry for total energy. Spin polarization was considered in all calculations.

Using this setup, we optimized the Gr lattice parameter, obtaining a value of 2.46 Å (corresponding to a C–C bond length of 1.42 Å), in agreement with previously reported values [58]. In addition, we analyzed the electronic properties to verify that the Dirac cone structure was accurately reproduced. To model Gr, a (6 × 7) supercell containing 84 carbon atoms was employed, with a single MPc molecule placed on the Gr sheet to form the MPc/Gr interface. Since MPc–MPc lateral interactions are negligible due to the apolar nature of the terminal C–H bonds, the interface is expected to be mainly governed by the interaction between MPc and Gr. The selected (6 × 7) Gr supercell provides a good balance: it is small enough to keep the computational cost low, while still being large enough to host a horizontally adsorbed MPc molecule without introducing significant distortion effects. Monkhorst–Pack [59] k-point grids of 6 × 6 × 1 and 18 × 18 × 1 were utilized for geometry optimization and density of states (DOS) calculations, respectively. A vacuum space of at least 20 Å was included in the supercell to prevent interactions between periodic images, along with a dipole correction to account for field effects.

To model the effect of charge doping in free-standing Gr, the calculations were performed by adding or removing a specific number of electrons (*e*) from the cell. Three doping conditions were considered: (1) a neutral system with no added or removed electrons, (2) an n-doped system with 0.89 *e* (≈0.011 *e*/Gr atom) added, and (3) a p-doped system with 0.89 *e* removed. The choice of this specific level of electron doping is intended to replicate the doping level observed in Gr-based systems. Indeed, a removal of ±0.89 e in our supercell model corresponds to a Dirac cone shift relative to the Fermi level of ≈ ±0.30 eV, which is in good agreement with both theoretical and experimental values of ≈ +0.30 eV (p-doped), ≈−0.30 eV (n-doped), and ≈−0.40 eV (n-doped) for Gr supported on Pt(111) [30], Cu(111) [30], and SiC(0001) [31], respectively.

The metal d-states were analyzed using the d-centroid descriptor [60], which was calculated based on the following formula:

$$d-\mathrm{centroid}=\frac{\int_{-\infty }^{+\infty }E\rho \left(E\right)dE}{\int_{-\infty }^{+\infty }\rho \left(E\right)dE}$$

where $\rho \left(E\right)$ denotes the d-projected density of states at the specific energy level, and E is the energy level (referenced to the Fermi energy).

The adsorption energies ($E_{ads}$) are calculated according to the following formula:

$$E_{ads}=\frac{E_{n,gas-MPc/Gr}-(E_{MPc/Gr}+nE_{gas})}{n}$$

where $E_{n,gas-MPc/Gr}$, $E_{MPc/Gr}$, and $E_{gas}$ represent the electronic energies of the optimized MPc/Gr system with adsorbed n gas molecules, the MPc/Gr system, and the isolated gas molecule, respectively.

## 3. Results and Discussion

### 3.1. Working Principle of a Graphene-Based Chemiresistor in Gas Sensing

**Figure 1:**
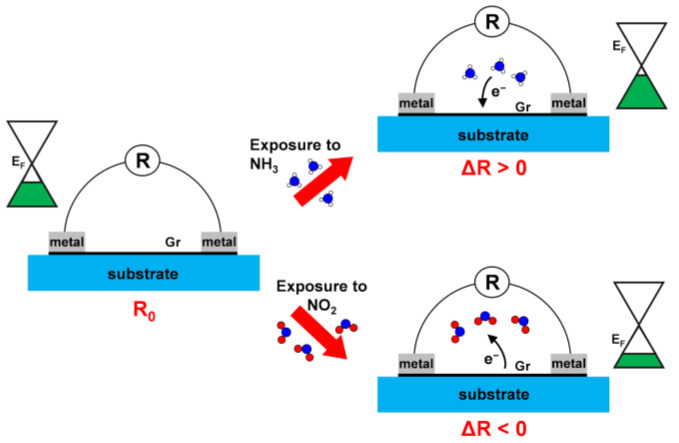
Schematic representation illustrating the operating configuration of a p-doped Gr-based chemiresistor device when exposed to NH_3_ (electron-donor gas) and NO_2_ (electron-acceptor gas).

Chemiresistor-based sensors are widely used for screening new gas-sensing materials due to their low cost and simple architecture [61], making them ideal for proof-of-concept experiments. In Gr-based chemiresistors, the setup consists of two metal electrodes contacting a graphene sheet, typically supported by an insulating substrate to maximize conductivity along the graphene layer (left panel of Figure 1). Applying a potential difference across the electrodes generates a baseline resistance (R_0_) in the Gr. Upon interaction with gas molecules, this resistance changes (ΔR), indicating the sensor’s response and sensitivity. A larger resistance shift at a given gas concentration corresponds to higher sensitivity. This shift is typically driven by changes in the charge carrier density: electron holes in p-type doped Gr (Figure 1) or electrons in n-type doped Gr.

Since, in these devices, the Gr layer is nearly decoupled from the insulating substrate, it essentially behaves as a free-standing layer, preserving its distinctive Dirac cone band structure (Figure 1). This unique feature ensures that the charge carrier density directly depends on the position of the Dirac cone minimum (E_D_), which is highly sensitive to any electron charge transfer between Gr and any external molecules or surfaces [33]. Even subtle charge transfers can induce measurable changes, making Gr an ideal platform for sensing applications [20].

In light of this, we will evaluate the response of functionalized Gr-based systems to probe gas molecules through a systematic approach. Section 3.2 delves into the interaction between MPc and Gr, with a particular focus on how MPc’s molecular orbitals couple with Gr’s electronic π states. Section 3.3 and Section 3.4 then examine the system’s response to two specific gases: NH_3_, an electron donor, and NO_2_, an electron acceptor. In particular, we assess how gas adsorption influences the charge carrier density in Gr, ultimately leading to a measurable resistance change, which serves as the sensing signal.

### 3.2. Influence of Metal Cation and Gr Charge State on the Electronic Properties of the MPc/Gr Interface

In our previous works [49,50], we developed a model describing the interface between p-doped Gr and NiPc molecules, in agreement with spectroscopic (X-ray Photoelectron Spectroscopy, XPS; Near Edge X-ray Absorption Fine Structure, NEXAFS) and microscopic (atomic force microscopy, AFM) experimental data. These studies demonstrate that NiPc molecules lie flat on the Gr surface, forming a complete monolayer. Building on this input, and assuming similar behavior for the other MPc molecules, we now investigate additional metal phthalocyanines (FePc, CoPc, and CuPc) and Gr charge states, ranging from p-doped to neutral and n-doped, to identify potential trends along the series.

**Figure 2:**
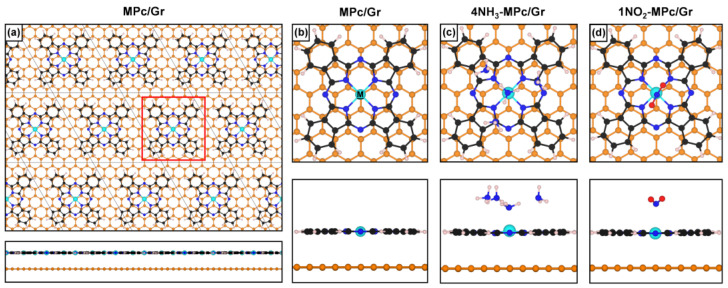
Ball-and-stick models of the optimized geometries for (**a**,**b**) MPc/Gr, (**c**) 4NH_3_-MPc/Gr, and (**d**) 1NO_2_-MPc/Gr. Color scheme: graphene C atoms are shown in orange; metal cations (M) in cyan; C, N, and H atoms in the MPc molecule are black, blue, and white, respectively; NH_3_ nitrogen and hydrogen atoms are blue and white; NO_2_ nitrogen and oxygen atoms are blue and red.

Each MPc/Gr system was fully relaxed, but the optimized geometries show minimal variation with changes in the metal cation or the Gr doping level. As shown in Figure 2a,b, the MPc molecules are horizontally oriented and uniformly distributed across the Gr surface, with a vertical distance of approximately 3.4–3.5 Å, depending on the specific MPc and Gr doping level (Table S1). We identified several nearly degenerate minimum-energy structures, differing in the stacking alignment of the molecule relative to the Gr layer. While some molecular mobility on Gr is expected at RT, we focus here on the configuration where the metal atom is positioned directly above a Gr carbon atom (Figure 2a,b). This specific alignment is likely to enhance the metal–graphene interaction, which is crucial for the system’s response to the target gas [50].

To investigate the influence of the metal cation on the MPc/Gr interaction, we first investigate the geometrical and electronic configurations of MPc molecules before adsorption on Gr.

From a geometrical perspective, MPc molecules exhibit a planar structure, with the metal cation coordinated by four N atoms (*pyrrolic*) from four distinct isoindole units, interconnected via N bridges (*aza*). The metal cation has an oxidation state of +2, while the macrocyclic organic ligand (Pc) acts as a divalent anion, resulting in an overall neutral molecule. The square planar coordination environment around M^2^⁺ is characterized by four equivalent M–N bonds, with our computed bond lengths in the range of approximately 1.9–2.0 Å, in agreement with previous theoretical studies [56,57].

**Figure 3:**
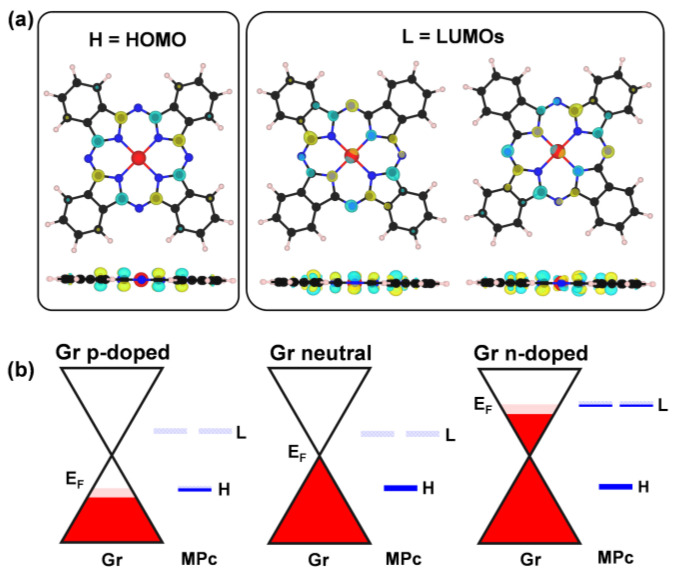
(**a**) Isosurface 3D plots (electron density threshold = 4 × 10^−3^ e^−^/bohr^3^) of the HOMO and LUMOs of gas-phase NiPc. Similar plots are obtained for the other transition metals (M = Fe, Co, Cu) and reported in Figure S1. Color scheme: Ni in red; C, N, and H atoms in black, blue, and white, respectively. (**b**) Energy alignment scheme of the frontier molecular orbitals (FMOs) of MPc with the π states of Gr upon forming the MPc/Gr interface. The Fermi level of the MPc/Gr system is indicated as E_F_. This scheme is derived from the computed projected density of states (PDOS) presented in Figure 5 (Gr p-doped), Figure S2 (Gr neutral), and Figure S3 (Gr n-doped).

From an electronic point of view, photoelectron spectroscopy (PES) studies on gas-phase samples or thin films of MPcs have demonstrated that the frontier molecular orbitals (FMOs) predominantly exhibit a π character delocalized over the porphyrazine ring [62,63,64,65]. These results are consistent with our calculations (Figure 3a and Figure S1) and agree well with previous studies based on *hybrid* DFT and DFT + U methods [56,57].

**Figure 4:**
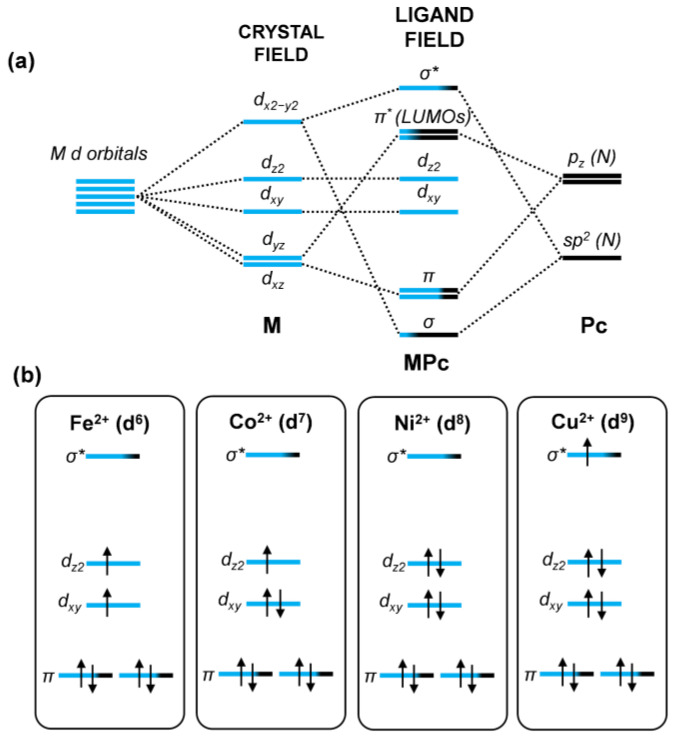
(**a**) Schematic representation of d-orbital splitting in a square planar crystal field and in a ligand field, showing the mixing with ligand σ and π orbitals. The newly formed mixed orbitals are labeled according to their type, with the color scale indicating the degree of orbital contribution. (**b**) Orbital occupations for the four metal cations are considered only for those orbitals with high metal contribution. For clarity, the energy levels of the different metal cations are fictitiously aligned. Electrons are represented by black arrows.

As shown in Figure 3a, the highest occupied molecular orbital (HOMO) is exclusively composed of contributions from the C p_z_ atomic orbitals, whereas the two degenerate lowest unoccupied molecular orbitals (LUMOs) also include p_z_ orbital contributions from the *aza* and *pyrrolic* N atoms and there is little contribution from the metal d-states (Figure 3a and Figure 4a).

**Figure 5:**
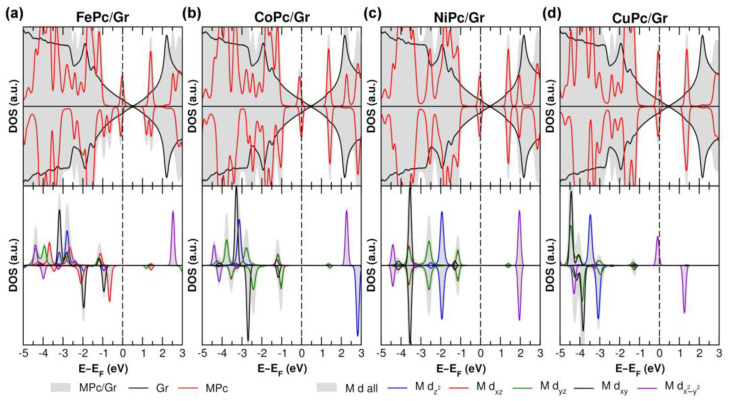
Total (TDOS) and projected (PDOS) density of states obtained using PBE + D3 + U for p-doped (**a**) FePc/Gr, (**b**) CoPc/Gr, (**c**) NiPc/Gr, and (**d**) CuPc/Gr. The top panels show the PDOS projected onto Gr and MPc states, while the bottom panels present the PDOS of the metal d-states. The color legend is displayed at the bottom, with the top panel legend positioned on the right and the bottom panel legend also on the right. The Fermi level is set to zero and indicated by a dashed line.

When the MPc molecule is deposited on a graphene layer, the HOMO/LUMOs couple with the Gr π states since they have compatible symmetries (see Figure 5, Figures S2 and S3 for the PDOS on MPc in the the neutral, n-doped, and p-doped cases, respectively). This coupling establishes charge transfer channels at the interface, enabling electron flow from Gr to MPc or vice versa, depending on the Gr charge state. For instance, as shown in Figure 3b, when Gr is positively charged (p-doped), electrons are transferred from the HOMO of MPc to Gr, leaving the HOMO partially occupied at the Fermi level. Conversely, when Gr is negatively charged (n-doped), it donates electrons to the LUMOs of MPc, which become partially filled. In the case of neutral Gr, where the Dirac point aligns with the Fermi level, no charge transfer is observed.

The question that arises is related to the position of the metal d orbitals. According to crystal field theory, when a metal center is in a square planar coordination, the five degenerate d orbitals undergo splitting (Figure 4a) according to the energy order, from lowest to highest: the degenerate d_xz_ and d_yz_ orbitals, d_xy_, d_z2_, and d_x2−y2_. However, the M–N coordination is better described using the ligand field theory, as additional covalent interactions beyond electrostatic effects arise due to the mixing with Pc MOs with σ and π symmetry. Consequently, the resulting MPc orbitals, according to the ligand field theory, have bonding (σ_dx2−y2_ and π_dxz_/π_dyz_), non-bonding (d_z2_ and d_xy_), and anti-bonding (σ_dx2-y2_* and π_dxz_*/π_dyz_*) character (Figure 4a). Their occupation will depend on the number of electrons in the metal center, ultimately determining the molecule’s spin multiplicity. In particular, in Figure 4b, we present the occupation of the orbitals with high metal d character according to the number of d electrons for a specific transition metal cation. The π_dxz_*/π_dyz_* results as always being empty and correspond to the LUMOs of the MPc, discussed above.

For instance, the ground state of FePc remains a subject of ongoing debate, both experimentally and computationally [66], with uncertainty persisting between a triplet and a quintet ground state. In the triplet configuration, two unpaired electrons occupy the non-bonding d_z2_ and d_xy_ orbitals, whereas in the quintet state, one π orbital, d_xy_, d_z2_, and σ* orbitals are now singly occupied. Our calculations, summarized in Table S2, indicate that the triplet configuration is the most energetically stable, followed by the quintet (+0.20 eV) and the singlet (+1.19 eV), where the two unpaired electrons pair up in the d_xy_. In contrast, there is consensus on the electronic structure of CoPc: the Co ion has one more electron with respect to the Fe ion, which results in a doublet ground state CoPc [56,67], with the unpaired electron in the d_z2_ orbital [56]. In NiPc, where Ni is in a d^8^ configuration, all d electrons are paired up [68], leaving the anti-bonding σ* orbital unoccupied. Our calculations confirm this analysis for CoPc and NiPc (Table S2).

Finally, in CuPc, since Cu has one more electron (d^9^), the σ* orbital becomes singly occupied, leading to a doublet ground state [68,69], as confirmed by our calculations (Table S2).

When the MPc molecule is deposited onto the Gr layer, the weak interaction that is established largely preserves the MO order (see Figure 5, Figures S2 and S3 for the PDOS on the metal center in the p-doped, neutral, and n-doped cases, respectively). As a consequence of the short distance between the MPc molecular plane and the Gr layer due to the π-π stacking interaction, only for the d orbitals with a z-component (i.e., d_xz_, d_yz_, and d_z2_), we observe some coupling with the Gr π states. This effect is evident when comparing the PDOS of the gas-phase and Gr-supported NiPc (Figure S4a): upon adsorption on Gr, the d_xz_, d_yz_, and d_z2_ orbitals exhibit both intensity reduction and increased broadening of the density of states, unlike the other d orbitals. Additionally, as shown in the 3D plot of the integrated local density of states (ILDOS) for the d_z2_ PDOS (Figure S4b), a clear spatial overlap is observed between the metal center and the Gr π states, further confirming their interaction.

Finally, to evaluate the relative energy stability of the MPc/Gr systems, we computed and compared the adsorption energies of different MPc molecules on the Gr surface with varying charge states. The results, summarized in Table S1, reveal a clear trend: as the doping level changes from p-type to neutral and subsequently to n-type, the adsorption energy becomes progressively less negative (from ≈−1.4 eV/nm^2^ to ≈−0.9 eV/nm^2^), indicating reduced interaction strength and stability. Among the series, FePc displays the strongest adsorption energy under all doping conditions, whereas the other MPc molecules exhibit similar binding.

### 3.3. Interaction with Electron-Donor Gas: NH_3_ Adsorption on MPc/Gr

After having prepared and characterized the MPc/Gr interface models, we now proceed with the investigation of their response to test gases, starting with an electron-donor species, i.e., NH_3_. In our previous work [50], we determined that a minimum of four NH_3_ molecules, arranged in a specific configuration, is required to induce a detectable response in the NiPc/Gr system. Therefore, in the following we consider the same four-molecule configuration. Specifically, one NH_3_ molecule orients its N sp^3^ lone pair (HOMO of NH_3_) towards the metal cation, while the remaining three act as H-bond acceptors (Figure 2c). This arrangement raises the energy of the HOMO in the H-donating molecule adsorbed on the MPc, facilitating electron charge transfer, through the metal atom, to the Gr layer. The corresponding energetics, based on the computed adsorption energies (E_ads_) of the NH_3_ clusters on the various MPc/Gr systems, are summarized in Table S3.

Considering p-doped NiPc/Gr as a representative system, the sequential adsorption of up to four NH_3_ molecules is energetically favorable, with E_ads_ values of −0.48 eV, −0.37 eV, −0.35 eV, and −0.37 eV for one to four molecules, respectively. The relative probabilities (P_i_), calculated via the Boltzmann distribution and reported in Table S4 along with computed recovery times (τ), indicate that the four-molecule configuration corresponds to a local minimum accessible under equilibrium conditions.

The adsorption of the first NH_3_ molecule (−0.48 eV) ensures a residence time long enough to allow for hydrogen bonding and stepwise cluster formation. This configuration is thus representative of a possible sensing-active state, even if its actual frequency under experimental conditions may be lower than that predicted by static models.

The estimated residence times at 300 K show that the first molecule remains adsorbed for ~10 μs, providing a stable nucleation site, while subsequent H-bonded molecules exhibit shorter lifetimes (0.1–0.2 μs). These timescales are sufficient for transient cluster formation and charge transfer to the Gr layer, which occurs on a femtosecond timescale.

**Figure 6:**
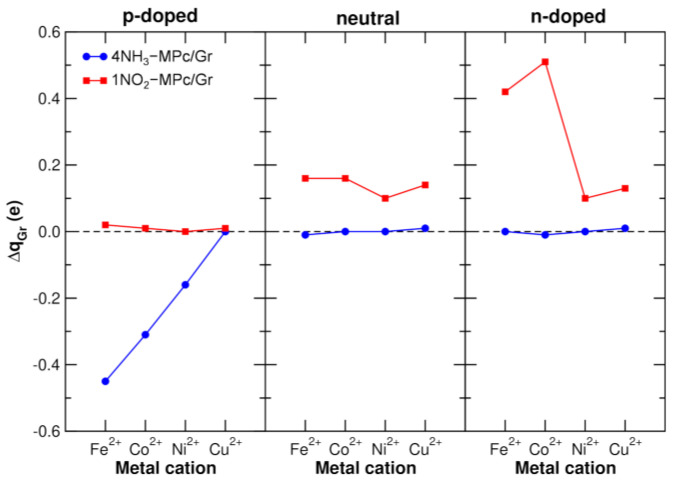
Atomic charge variation (where Δq_Gr_ = q_Gr,gas−MPc/Gr_ − q_Gr,MPc/Gr_) calculated using the Bader scheme, summed over all graphene carbon atoms for the various MPc/Gr systems after gas adsorption. Each panel represents a different charge condition: p-doped (left), neutral (middle), and n-doped (right). The x-axis indicates the different MPc systems considered, while the blue and red lines correspond to the charge variation upon NH_3_ and NO_2_ adsorption, respectively.

A straightforward method to assess the sensitivity of different MPc/Gr systems to NH_3_ is to compute the charge variation in Gr, which reflects changes in the charge carrier density before and after NH_3_ exposure. In Figure 6 (blue points and lines), we present the charge variation on the Gr layer (where Δq_Gr_ = q_Gr,4NH3-MPc/Gr_ − q_Gr,MPc/Gr_), calculated using the Bader partition scheme [70], for each considered doping level: p-doped, neutral, and n-doped. When Δq_Gr_ = 0, there is no charge transfer to Gr, while a positive/negative value implies that Gr is losing/gaining electrons. Any charge transfer will affect the charge carrier density in the Gr layer. For instance, in the p-doped case, some electron transfer to Gr reduces the number of holes (the majority charge carriers), resulting in an increase in resistance. Conversely, in the n-doped case, some electron transfer to Gr increases the number of electrons (the majority charge carriers), leading to a decrease in resistance. Thus, the resistance change is directly linked to the doping level and the nature of charge transfer, providing valuable insights into the sensing capabilities of the system.

Notably, upon NH_3_ exposure, a change in the Gr total charge is observed only in the p-doped case (left panel), whereas the neutral (middle panel) and n-doped (right panel) cases exhibit negligible charge variations. This suggests that the electronic interaction between NH_3_ and Gr is highly dependent on the Gr doping level.

In the view on this, a closer examination of the p-doped case reveals a clear trend within the MPc series. As shown in Figure 6, in the p-doped case, FePc exhibits the highest Δq_Gr_ value, with a gradual decrease along the series, reaching CuPc, where no charge variation is detected. This indicates that the CuPc/Gr system is not sensitive to NH_3_.

Based on these results, two key questions arise: (1) why is a p-doping condition necessary to achieve a response to NH_3_? And (2) why does the response follow the trend FePc/Gr > CoPc/Gr > NiPc/Gr, with CuPc/Gr failing to show any response?

**Figure 7:**
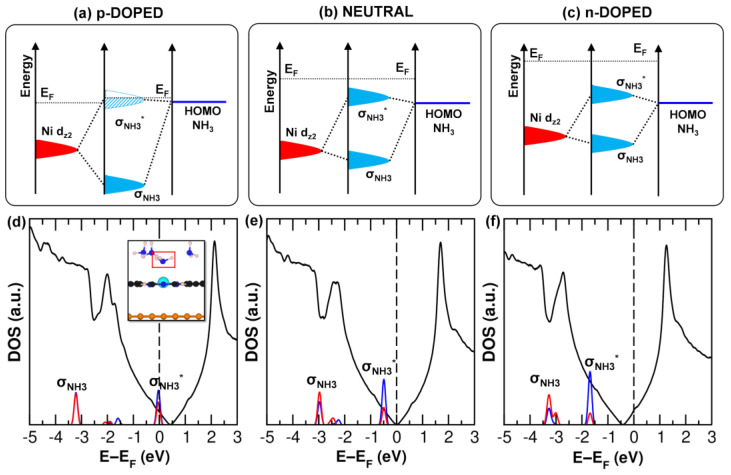
(**a**–**c**) Schematic representation of the orbital mixing for the 4NH_3_-NiPc/Gr system (as shown in Figure 2c) in the p-doped (**a**), neutral (**b**), and n-doped (**c**) cases. The orbital alignment in the schematic representation is based on the projected density of states (PDOS) for Gr (black curve), the NH_3_ molecule placed on top of Ni (blue curve), and the Ni d_z2_ orbital (red curve), as shown in (**d**–**f**) for the interacting system, and on the alignment with respect to the vacuum level for each non-interacting fragment (NH_3_ and NiPc/Gr).

To address them, we must recall the sensing mechanism recently proposed for p-doped NiPc/Gr [50], in which the charge transfer from NH_3_ to Gr is mediated by the Ni d_z2_ orbital. This orbital is particularly relevant due to its dual nature: it is non-bonding and localized on the metal site, yet it also couples with the Gr π states (as discussed in the previous section and shown in Figure S4b), enabling NH_3_ to affect the Gr charge carriers. Specifically, upon interaction with the gas molecule, a new set of bonding (σ) and anti-bonding (σ*) orbitals forms through the mixing of the Ni d_z2_ with the N sp^3^ lone pair (HOMO) of the Ni-coordinated NH_3_ (Figure 7a–c). If the anti-bonding orbital lies above the Fermi level of the MPc/Gr system (essentially, the Fermi level of Gr), there will be a driving force for electrons to move to Gr, leading to an observable charge variation.

For the sake of clarity, the following analysis will focus on the NiPc/Gr case, as it is the simplest system due to the absence of spin polarization. However, apart from CuPc/Gr, the behavior of FePc/Gr and CoPc/Gr follows a similar trend to the one we will present for NiPc/Gr.

In Figure 7a–c, we present the schematic representation of the orbital alignment before and after mixing upon NH_3_ adsorption at different doping levels. The three qualitative pictures are based on the calculated project DOS (PDOS) of Gr, the coordinating NH_3_ molecule, and the Ni d_z2_ orbital in Figure 7d–f. Going from the p-doped to neutral and to n-doped Gr, the Fermi level becomes higher in energy from the HOMO of the gas-phase NH_3_ (Figure 7a–c). Once the NH_3_ adsorbs on the metal atom, the resulting σ* state is sufficiently high in energy to allow charge transfer to Gr only in the case of p-type doped Gr, which gains electronic charge from NH_3_. In contrast, for the n-doped and neutral cases, the σ* state is far below the Fermi level. This analysis addresses question (1) above.

**Figure 8:**
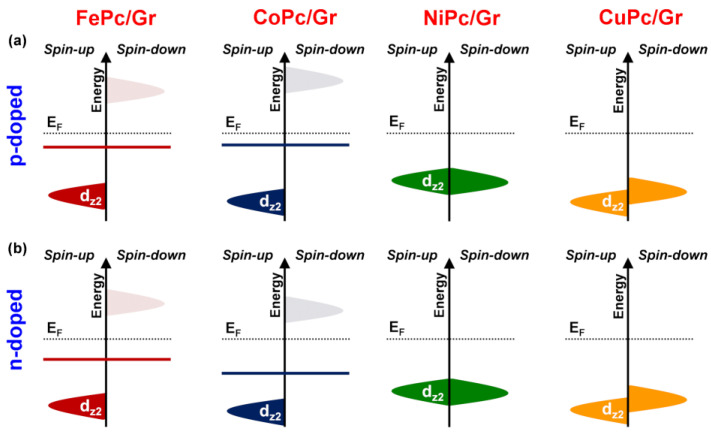
Energy alignment of the metal d_z2_ orbital for the four (**a**) p-doped and (**b**) n-doped MPc/Gr interfaces. All systems are aligned with respect to the Fermi level (E_F_), with spin-up and spin-down components shown separately on the left and right sides, respectively. Occupied and unoccupied orbitals are represented by solid and dashed areas, respectively. For FePc/Gr and CoPc/Gr, the d_z2_ centroid is indicated by a solid line, while for NiPc/Gr and CuPc/Gr, the centroid corresponds to the spin-up and spin-down orbital components due to negligible or absent spin polarization on the metal d-states and is not explicitly shown.

Based on the proposed charge transfer mechanism above that was illustrated for Ni and successful only for the p-type doped Gr, we now examine the other metals to address question (2). The descriptor we focus our attention on is the position of the d_z2_ orbital that is involved in the interaction with both Gr and NH_3_ (to form the σ_NH3_* state). Figure 8a schematically illustrates the relative position of this d_z2_ orbital, for the four metal atoms under investigation, with respect to the Fermi level of Gr. A direct comparison is not straightforward, as in Fe and Co, the d_z2_ orbital is spin-unbalanced—the spin-up component is filled and the spin-down component is empty—while in Ni and Cu, the d_z2_ orbital is fully occupied. To solve this issue, we computed the d_z2_-state centroid (Table S2), as proposed in previous studies [71]. The centroid is shown only for the spin-unbalanced cases as a horizonal segment. We observe a progressive stabilization further from the Fermi level going from Fe to Cu, along the series. A d_z2_ centroid closer to the Fermi level will also be closer in energy to the HOMO of NH_3_ (Figure 7a), facilitating the orbital mixing. A larger metal/NH_3_ interaction results in a larger σ_NH3_/σ_NH3_^*^ and, thus, in a higher position of the σ_NH3_* state, which favors charge transfer to Gr (Figure 7a).

However, there is an exception in the case of Fe. The centroid position of Fe is further below the Fermi level than that of Co, despite a larger charge (0.45 e vs. 0.30 e for Fe vs. Co, respectively). This discrepancy from the proposed rationalization can be explained by the fact that NH_3_ coordination induces a spin configuration change in the metal center, stabilizing the Fe high-spin quintet state, in contrast to the triplet state observed in the absence of the adsorbed gas. In all other metal cases, gas adsorption does not alter the spin multiplicity.

Since FePc/Gr exhibits the highest sensitivity to NH_3_, we investigated its response to a single gas molecule, corresponding to a concentration four times lower than previously considered. This represents the limit at which a single gas molecule can be detected per deposited MPc molecule. In our previous work [50], we found that NiPc/Gr is not sensitive to a single NH_3_ molecule. Surprisingly, however, FePc/Gr can detect even a single NH_3_ molecule, which donates 0.07 *e* to Gr, altering its resistive response.

Finally, to evaluate the sensor’s recovery performance, as performed in previous studies [13,72], we calculated the recovery times (τ) at T = 300 K for the systems responsive to NH_3_, as shown in Figure 6. According to the values in Table S5, the recovery times range from 4 µs to 251 µs. This is short enough to prevent sensor poisoning at RT while still allowing sufficient time for charge transfer to the Gr layer, which occurs on the femtosecond scale.

### 3.4. Interaction with Electron-Acceptor Gas: NO_2_ Adsorption on MPc/Gr

We now investigate the response to an acceptor gas like NO_2_. Our previous findings [41] indicate that a single molecule is sufficient to trigger a response in p-doped NiPc/Gr; therefore, we consider this concentration here. As shown in Figure 2d, for all metals in the optimized structure, NO_2_ adsorbs above the metal cation in an N-down configuration. The corresponding energetics, based on the computed E_ads_ of NO_2_ on the various MPc/Gr systems, are reported in Table S3.

To assess the impact of Gr’s doping level on the sensitivity, we analyze the charge variation upon NO_2_ adsorption (red points in Figure 6), following the same approach as for NH_3_. However, unlike NH_3_, where only the p-doped system exhibited a response, NO_2_ shows no charge transfer in the p-doped case. Instead, a positive Δq_Gr_, indicating electron depletion from Gr, is observed in the neutral and n-doped cases. The largest variation occurs in the n-doped case, with FePc and CoPc displaying the highest sensitivity. Notably, this broader charge response range contrasts with the NH_3_ case, where sensitivity was confined to a specific doping condition (p-type).

**Figure 9:**
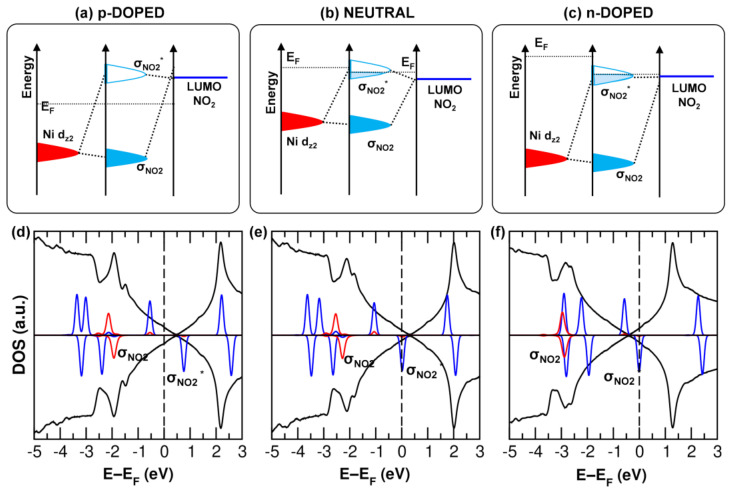
(**a**–**c**) Schematic representation of the orbital mixing for the NO_2_-NiPc/Gr system (as shown in Figure 2d) in the p-doped (**a**), neutral (**b**), and n-doped (**c**) cases. The orbital alignment in the schematic representation is based on the projected density of states (PDOS) for Gr (black curve), the NO_2_ molecule (blue curve), and the Ni d_z2_ orbital (red curve), as shown in (**d**–**f**) for the interacting system, and on the alignment with respect to the vacuum level for each non-interacting fragment (NO_2_ and NiPc/Gr).

To explain these trends, we can refer to similar arguments used for the NH_3_ case, but with key differences. Here, the charge transfer requires filling the LUMO of NO_2_ with electrons from Gr. In the p-doped MPc/Gr system, the Fermi level is positioned below the NO_2_ LUMO, preventing any charge transfer (Figure 9a). Conversely, in the neutral and n-doped cases, the Fermi level lies above the LUMO, enabling electron transfer (Figure 9). This process is facilitated by orbital coupling, where the metal d_z2_ orbital once again plays a crucial role.

The stronger response observed for FePc/Gr and CoPc/Gr can be attributed to the higher position of their metal d_z2_ centroid (Figure 8b). Since it lies closer to the NO_2_ LUMO, it allows for better orbital coupling, facilitating electron transfer from Gr to the gas molecule and enhancing the overall sensitivity.

Finally, similarly to the case of NH_3_, we computed the recovery times for NO_2_ desorption (Table S5). Under neutral conditions, the recovery times range from 1 ns to 1 µs, ensuring rapid desorption while still allowing sufficient time for charge transfer, which typically occurs on the femtosecond scale. In contrast, under n-doped conditions, the FePc/Gr and CoPc/Gr systems exhibit longer recovery times, suggesting that higher temperatures may be required to achieve full recovery.

## 4. Conclusions

In this work, we investigated the structural and electronic properties of MPc/Gr interfaces and their interactions with NH_3_ and NO_2_ using PBE + U + D3 calculations. We explored how different metal cations (Fe, Co, Ni, and Cu) and Gr doping levels (p-doped, neutral, and n-doped) influence interfacial interactions with target gases, aiming to enhance gas sensitivity.

Our analysis of MPc adsorption on Gr revealed nearly degenerate horizontally flat configurations with varying stacking arrangements. Electronically, MPcs retain their gas-phase orbital configurations and spin multiplicity, while their out-of-plane orbitals (HOMO, LUMO, and metal d_z2_) couple to the Gr π states, enabling interfacial charge transfer. Depending on the Gr charge state, electrons transfer either from MPc to Gr or vice versa. In the p-doped case, charge transfer reduces Gr’s hole concentration, whereas in the n-doped case, Gr donates electrons to MPc, becoming less electron-rich. Neutral Gr remains largely unaffected.

We then examined gas adsorption and its impact on the electronic properties of MPc/Gr. NH_3_ and NO_2_ act as electron donor and acceptor molecules, respectively, modifying Gr’s charge carrier density by altering the hole concentration in p-doped conditions and electron concentration in n-doped conditions.

Based on our calculations, the electron charge transfer from the gas molecule (NH_3_ or NO_2_) to the Gr layer is mediated by the metal d_z2_ orbital of the MPc molecule through its mixing with the frontier molecular orbitals of the gases: the HOMO of NH_3_ (electron-donor gas) and the LUMO of NO_2_ (electron-acceptor gas). This orbital mixing enables the electron transfer, as the d_z2_ orbital is coupled with the Gr π states.

For NH_3_, charge transfer occurs only under p-doping, regardless of the MPc type. The extent of transfer varies, with FePc showing the highest sensitivity, followed by CoPc, NiPc, and finally CuPc, which remains insensitive. This trend is dictated by the position of the metal d_z2_ orbital, which facilitates interaction between the NH_3_ HOMO and the Gr π-accepting states. Under neutral and n-doped conditions, despite orbital mixing, charge transfer is suppressed due to energy misalignment. The sensitivity trend follows the order CuPc < NiPc < CoPc < FePc, in line with the progressive shift of the d_z2_ orbital closer to the Fermi level. Remarkably, FePc/Gr exhibits sensitivity down to the limit of a single NH_3_ molecule per MPc.

For NO_2_, both neutral and n-doped conditions enable sensitivity, with the highest response observed for n-doped FePc/Gr and CoPc/Gr. Here, NO_2_ requires its empty LUMO to be filled via charge transfer from Gr. The higher position of the metal d_z2_ orbital in Fe and Co enhances this process. Additionally, since the NO_2_ LUMO is spin-polarized, the spin polarization of the Fe d_z2_ and Co d_z2_ orbitals further strengthens orbital mixing and charge transfer.

In conclusion, we demonstrated that the choices of metal cation and Gr doping are key parameters for optimizing the gas sensitivity of MPc/Gr in chemiresistive devices. We identified the best metal/doping combinations to enhance sensitivity toward electron-donating and electron-accepting gases. These findings offer valuable insights for the design of MPc/Gr-based sensors, which require high sensitivity, fast response, and quick recovery times for detecting gases such as NH_3_ and NO_2_ at RT, with potential applications in advanced technological fields such as environmental monitoring, food quality tracking, and breathomics.

## Data Availability

The raw data supporting the conclusions of this article will be made available by the authors on request.

## Supplementary Materials

The following supporting information can be downloaded at https://www.mdpi.com/article/10.3390/nano15090691/s1. Table S1: Adsorption energies and MPc-Gr vertical distances for MPc on Gr at different Gr doping levels; Table S2: Relative energies for different spin multiplicity configurations of FePc, CoPc, NiPc, and CuPc in the gas phase; Table S3: N–M distances between the metal center and the coordinated NH₃ or NO₂ molecules, and corresponding gas adsorption energies for various MPc/Gr systems, as shown in Figure 2c,d of the main text; Table S4: Adsorption energies for the sequential adsorption of one to four NH_3_ molecules on p-doped NiPc/Gr, along with the corresponding relative populations calculated using the Boltzmann distribution and recovery times; Table S5: Adsorption energies for selected systems showing response to the tested gases, along with the corresponding recovery times; Table S6: d_z2_-band centroid values for p-doped MPc/Gr, presented for the spin-up, spin-down, and combined (spin-up + spin-down) components; Figure S1: Total (TDOS) and projected (PDOS) density of states computed using PBE-D3+U for (a) FePc, (b) CoPc, (c) NiPc, and (d) CuPc in the gas phase; Figure S2: Total (TDOS) and projected (PDOS) density of states obtained using PBE-D3+U for neutral (a) FePc/Gr, (b) CoPc/Gr, (c) NiPc/Gr, and (d) CuPc/Gr; Figure S3: Total (TDOS) and projected (PDOS) density of states obtained using PBE-D3+U for n-doped (a) FePc/Gr, (b) CoPc/Gr, (c) NiPc/Gr, and (d) CuPc/Gr; Figure S4: (a) Comparison of the projected density of states (PDOS) onto the Ni d-states computed using PBE-D3+U for gas-phase NiPc and p-doped NiPc/Gr. (b) Corresponding integrated local density of states (ILDOS), illustrating the coupling between the Ni d_z2_ and graphene π states.
