# Supplementary material for: Tailoring Metal Phthalocyanine/Graphene Interfaces for Highly Sensitive Gas Sensors
Source: Nanomaterials (Basel). 2025 May 3;15(9):691. doi: 10.3390/nano15090691 (PMC12074356; doi:10.3390/nano15090691)
Supplement: Supplementary file 1 [file nanomaterials-15-00691-s001.zip › nanomaterials-3576263-supplementary.pdf]

SUPPLEMENTARY MATERIALS

Tailoring Metal Phthalocyanine/Graphene Interfaces for Highly Sensitive Gas Sensors

Daniele Perilli,[a,*] Alberto Maria Rizzi,[a] and Cristiana Di Valentin[a,*]

[a] Department of Materials Science, University of Milano-Bicocca, via R. Cozzi 55, I-20125 Milano, Italy

[*]Correspondence: daniele.perilli@unimib.it, cristiana.divalentin@unimib.it

Table S1: Adsorption energies (in eV/nm²) and MPc-Gr vertical distances (in Å) for MPc on Gr at different Gr doping levels. The values are calculated as $\Delta E = (E_{MPc/Gr} - (E_{MPc} + E_{Gr}))/(\text{Gr surface area})$, where $E_{MPc/Gr}$, E_{MPc}, and E_{Gr} represent the electronic energies of the optimized MPc/Gr interface, the gas-phase MPc molecule, and the Gr surface, respectively. ΔE values are normalized per the surface area of the Gr layer (2.55 nm²).

	p-doped		*neutral*		*n-doped*	
	d MPc-Gr (Å)	ΔE (eV/nm^2)	d MPc-Gr (Å)	ΔE (eV/nm^2)	d MPc-Gr (Å)	ΔE (eV/nm^2)
FePc/Gr	3.46	-1.86	3.47	-1.44	3.44	-1.22
CoPc/Gr	3.46	-1.27	3.36	-1.00	3.45	-0.75
NiPc/Gr	3.42	-1.26	3.44	-1.00	3.44	-0.76
CuPc/Gr	3.44	-1.27	3.41	-1.00	3.39	-0.78

Table S2: Relative energies (in eV) for different spin multiplicity configurations of FePc, CoPc, NiPc, and CuPc in the gas phase. For each MPc, the energy is given relative to the most stable configuration.

	**d electrons configuration**	**Spin multiplicity**	**ΔE (eV)**
FePc	d^6	Singlet	+1.19
		Triplet	0.00
		Quintet	+0.20
CoPc	d^7	Doublet	0.00
		Quartet	+1.27
NiPc	d^8	Singlet	0.00
		Triplet	+1.25
CuPc	d^9	Doublet	0.00
		Quartet	+1.21

Table S3: N–M distances (in Å) between the metal center and the coordinated NH_3 or NO_2 molecules, and corresponding gas adsorption energies (in eV) for various MPc/Gr systems, as shown in Figure 2c–d of the main text. For NH_3, adsorption energies are normalized per single molecule. Energies were calculated using the formula provided in the Computational Methods section.

		**NH_3**		**NO_2**	
		**d N-M (Å)**	**E_{ads} (eV/NH_3)**	**d N-M (Å)**	**E_{ads} (eV/NO_2)**
p-doped	FePc/Gr	2.13	-0.41	2.81	-0.08
	CoPc/Gr	2.09	-0.50	2.01	-0.43
	NiPc/Gr	2.27	-0.39	2.87	-0.21
	CuPc/Gr	2.22	-0.42	2.89	-0.22
neutral	FePc/Gr	2.23	-0.41	2.15	-0.36
	CoPc/Gr	2.24	-0.38	2.09	-0.32

	NiPc/Gr	2.51	-0.25	2.83	-0.22
	CuPc/Gr	2.31	-0.33	2.81	-0.27
n-doped	FePc/Gr	2.18	-0.38	2.22	-1.10
	CoPc/Gr	2.30	-0.50	2.21	-1.11
	NiPc/Gr	2.94	-0.23	2.87	-0.71
	CuPc/Gr	2.39	-0.24	2.73	-0.78

Table S4: Adsorption energies (E_{ads}) for the sequential adsorption of one to four NH_3 molecules on p-doped NiPc/Gr, along with the corresponding relative populations calculated using the Boltzmann distribution ($P_i \propto e^{-\frac{E_{ads}}{K_B T}}$) and recovery times ($\tau = \upsilon^{-1} e^{-\frac{E_{ads}}{K_B T}}$), where ν is the attempt frequency (set to 10^{12} s^{-1}), K_B is the Boltzmann constant, and T is the temperature (set to 300 K). Each E_{ads} value represents the energy gain associated with the addition of one NH_3 molecule, that is, from 0 to 1, 1 to 2, 2 to 3, and 3 to 4 molecules adsorbed.

**no. NH_3**	**E_{ads} (eV)**	**P_i**	**τ (s)**
1	-0.48	1.16×10^{8}	1.16×10^{-4}
2	-0.37	1.64×10^{6}	1.64×10^{-6}
3	-0.35	7.59×10^{5}	7.59×10^{-7}
4	-0.37	1.64×10^{6}	1.64×10^{-6}

Table S5: Adsorption energies (E_{ads}) for selected systems showing response to the tested gases, along with the corresponding recovery times. Recovery times are calculated using the Arrhenius-like expression $\tau = \upsilon^{-1} e^{-\frac{E_{ads}}{K_B T}}$, where ν is the attempt frequency (set to 10^{12} s^{-1}), K_B is the Boltzmann constant, and T is the temperature (set to 300 K).

		**E_{ads} (eV)**	**τ (s)**
p-doped	$4NH_3$-FePc/Gr	-0.41	7.73×10^{-6}
	$4NH_3$-CoPc/Gr	-0.50	2.51×10^{-4}
	$4NH_3$-NiPc/Gr	-0.39	3.93×10^{-6}
Neutral	$1NO_2$-FePc/Gr	-0.36	1.12×10^{-6}
	$1NO_2$-CoPc/Gr	-0.32	2.38×10^{-7}
	$1NO_2$-NiPc/Gr	-0.22	4.97×10^{-9}
	$1NO_2$-CuPc/Gr	-0.27	3.44×10^{-8}
n-doped	$1NO_2$-FePc/Gr	-1.10	3.02×10^{6}
	$1NO_2$-CoPc/Gr	-1.11	4.45×10^{6}
	$1NO_2$-NiPc/Gr	-0.71	8.47×10^{-1}

	1NO_2-CuPc/Gr	-0.78	1.27

Table S6: d_{z2}-band centroid values (in eV) for p-doped MPc/Gr, presented for the spin-up, spin-down, and combined (spin-up + spin-down) components. These values were computed using the formula described in the Computational Details section.

		**Spin up (eV)**	**Spin down (eV)**	**d_{z2}-band centroid (eV)**
p-doped	FePc	-3.76	+2.32	-0.72
	CoPc	-3.96	+2.64	-0.66
	NiPc	-2.58	-2.58	-2.58
	CuPc	-4.18	-3.68	-3.93
n-doped	FePc	-4.84	+1.98	-1.43
	CoPc	-5.26	+1.28	-1.99
	NiPc	-3.91	-3.91	-3.91
	CuPc	-5.01	-4.65	-4.83

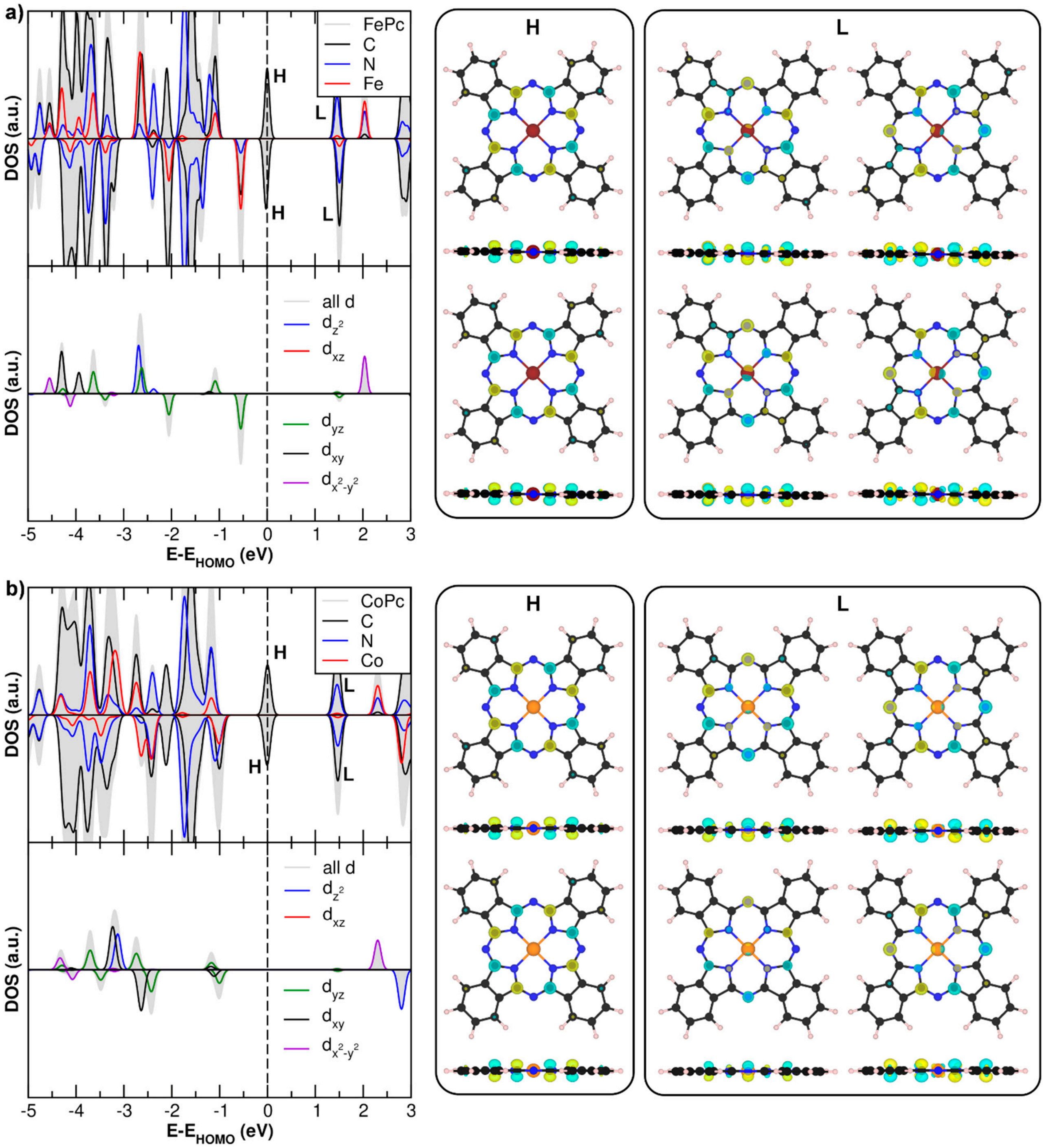
a)
FePc
C
N
Fe
H
L
DOS (a.u.)
all d
d_{z^2}
d_{xz}
d_{yz}
d_{xy}
$d_{x^2-y^2}$
-5 -4 -3 -2 -1 0 1 2 3
$E-E_{HOMO}$ (eV)
b)
CoPc
C
N
Co
H
L
DOS (a.u.)
all d
d_{z^2}
d_{xz}
d_{yz}
d_{xy}
$d_{x^2-y^2}$
-5 -4 -3 -2 -1 0 1 2 3
$E-E_{HOMO}$ (eV)

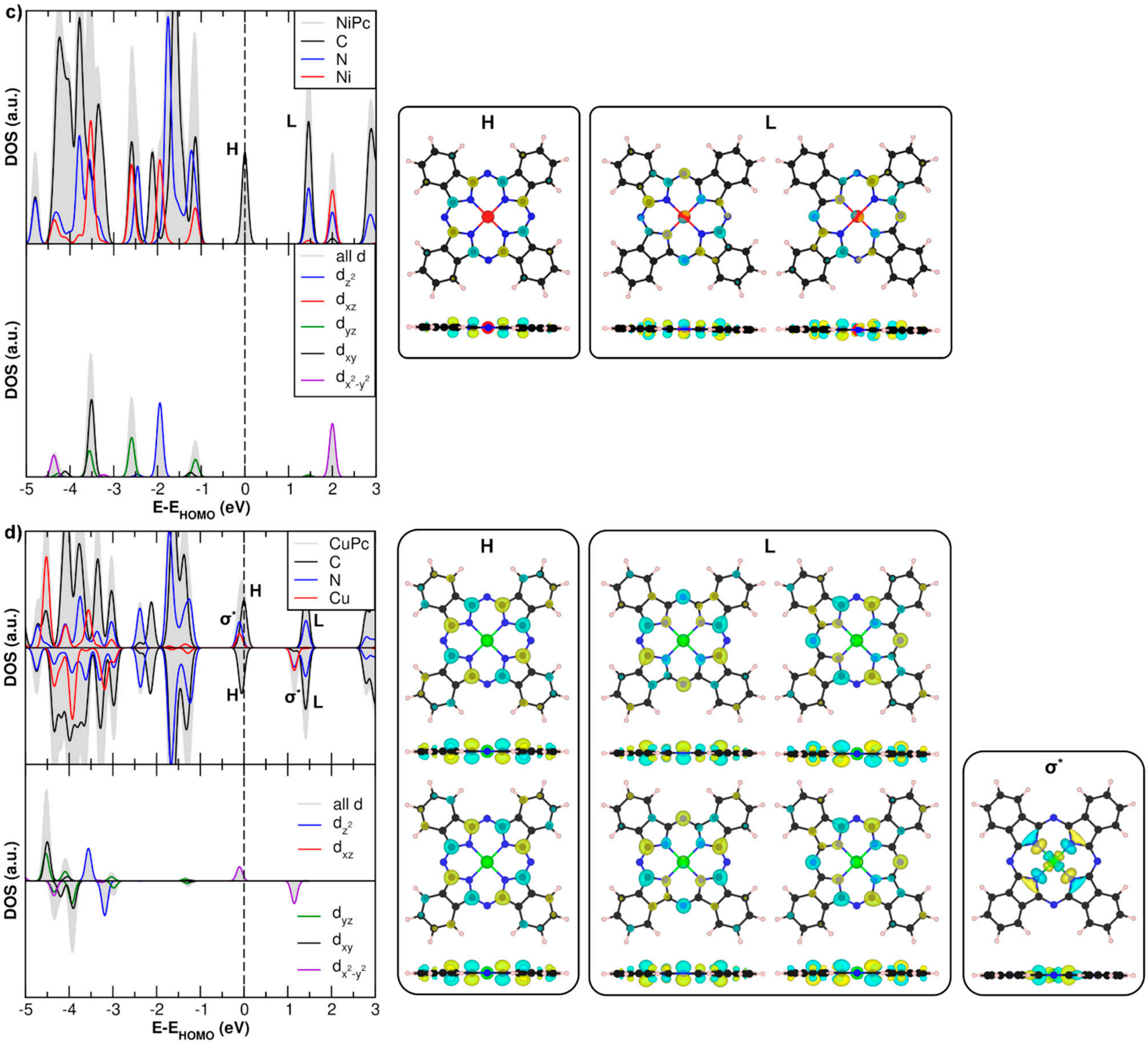

Figure S1: Total (TDOS) and projected (PDOS) density of states computed using PBE-D3+U for (a) FePc, (b) CoPc, (c) NiPc, and (d) CuPc in the gas phase. The top-left panels display the TDOS (gray area) and PDOS projected onto C, N, and M states, while the bottom-left panels focus on the PDOS of the metal d-states. The color legend is provided within each panel. The HOMO level is set to zero and marked by a dashed line. The right panels present isosurface 3D plots of the HOMO, LUMOs, and σ* states (denoted as H, L, and σ*), with an electron density threshold of 2×10^{-3} $e^-/bohr^3$ for Fe, Co, and Cu, and 4×10^{-3} $e^-/bohr^3$ for Ni. The top and bottom plots correspond to the spin-up and spin-down components, respectively. Notably, in CuPc, the σ* state is singly occupied, causing its empty spin-down component to be lower in energy than the LUMOs, unlike the other metals.

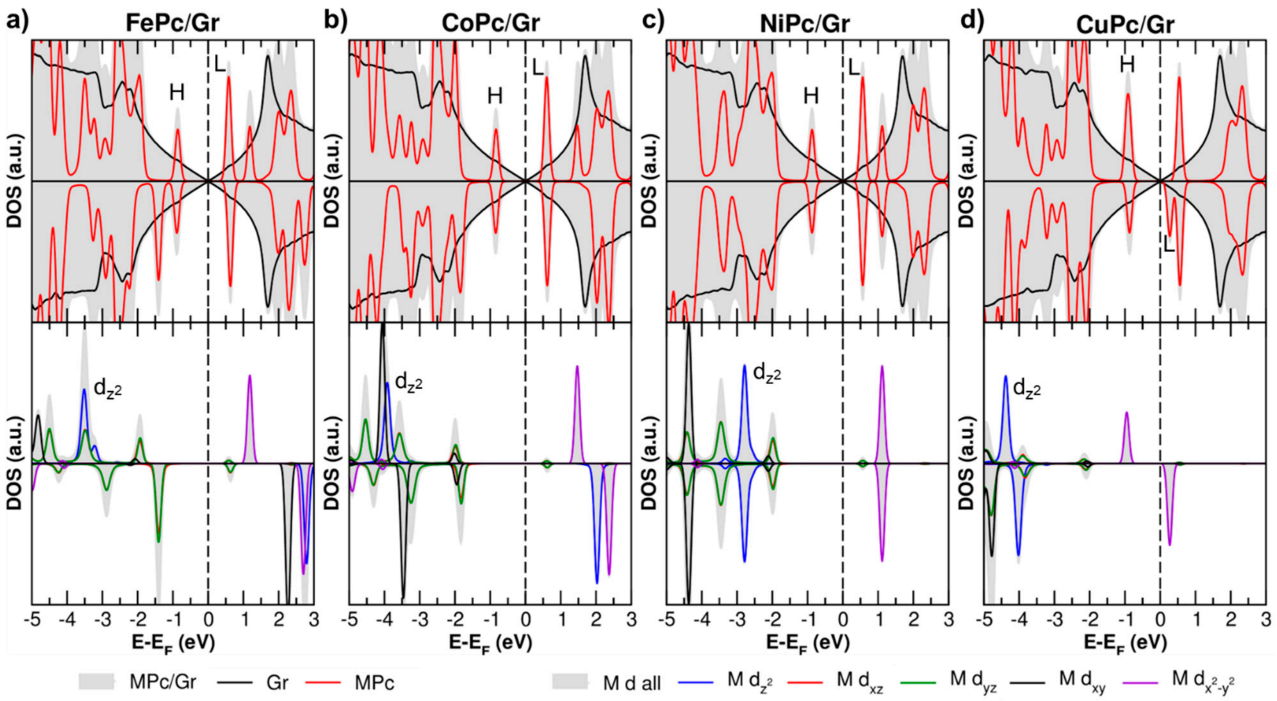

Figure S2: Total (TDOS) and projected (PDOS) density of states obtained using PBE-D3+U for neutral (a) FePc/Gr, (b) CoPc/Gr, (c) NiPc/Gr, and (d) CuPc/Gr. The top panels show the PDOS projected onto Gr and MPc states, while the bottom panels present the PDOS of the metal d-states. The color legend is displayed at the bottom, with the top panel legend positioned on the right and the bottom panel legend also on the right. The Fermi level is set to zero and indicated by a dashed line.

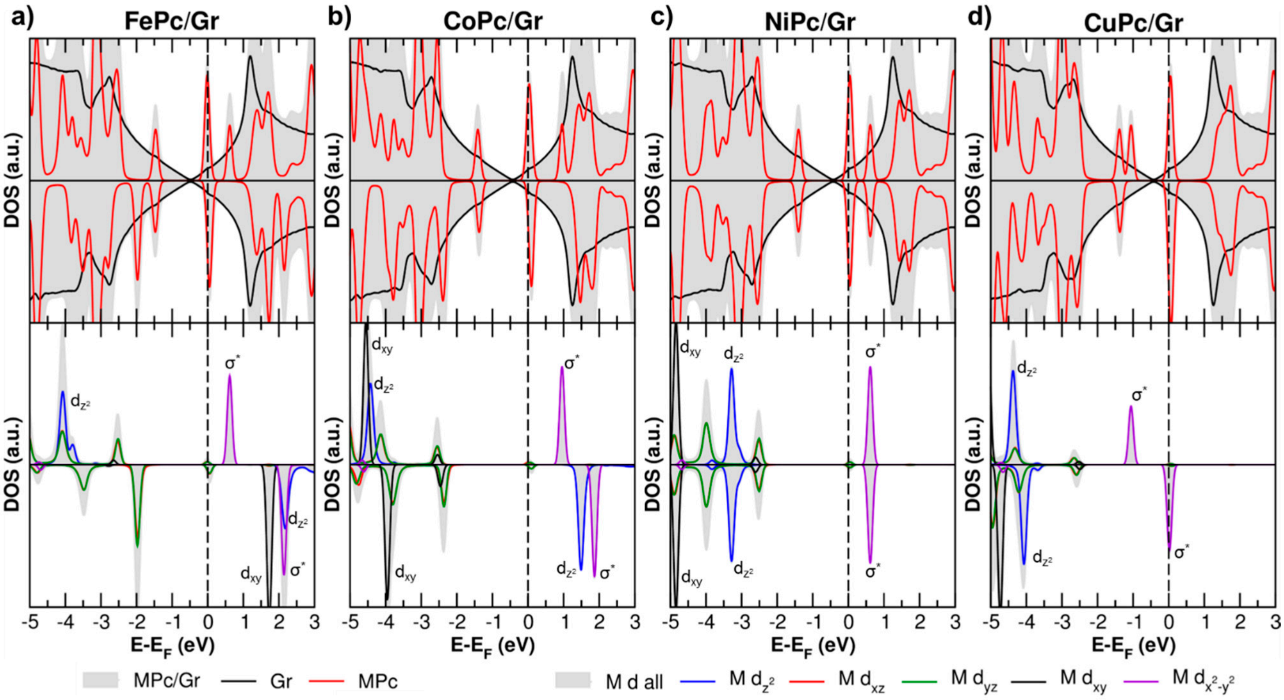

Figure S3: Total (TDOS) and projected (PDOS) density of states obtained using PBE-D3+U for n-doped (a) FePc/Gr, (b) CoPc/Gr, (c) NiPc/Gr, and (d) CuPc/Gr. The top panels show the PDOS projected onto Gr and MPc states, while the bottom panels present the PDOS of the metal d-states.

The color legend is displayed at the bottom, with the top panel legend positioned on the right and the bottom panel legend also on the right. The Fermi level is set to zero and indicated by a dashed line.

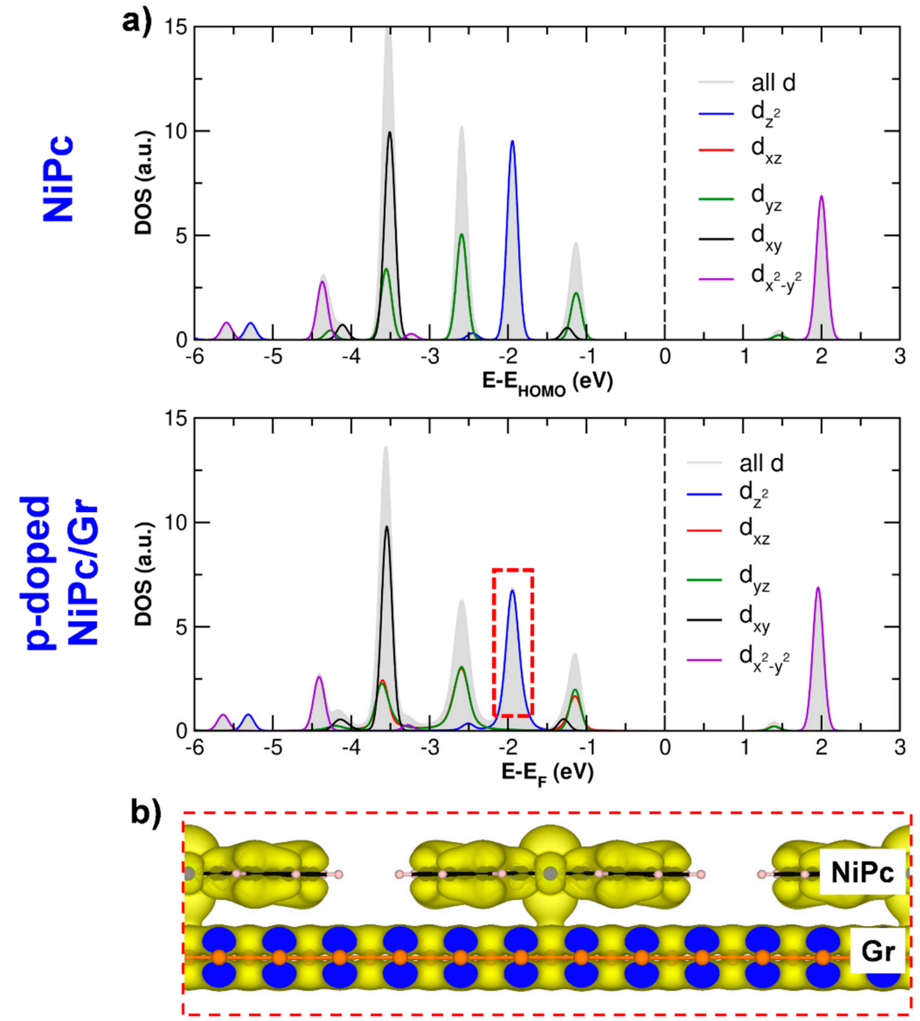

Figure S4: (a) Comparison of the projected density of states (PDOS) onto the Ni d-states computed using PBE-D3+U for gas-phase NiPc and p-doped NiPc/Gr. The PDOS of the Ni d_{z2} state in the Gr-supported system is highlighted with a red dashed box. (b) Corresponding integrated local density of states (ILDOS), illustrating the coupling between the Ni d_{z2} and graphene π states, as indicated by the red dashed box. The electron density threshold is set to 1×10^{-3} $e^-/bohr^3$.
